# MOG-antibody-associated transverse myelitis with the H-sign and unusual MRI enhancement: a case report and literature review

**DOI:** 10.3389/fped.2024.1451688

**Published:** 2024-09-10

**Authors:** Lu Zhang, Chuan Feng, Ling He, Shi-Yu Huang, Xin-Yin Liu, Xiao Fan

**Affiliations:** Department of Radiology, National Clinical Research Center for Child Health and Disorders, Ministry of Education Key Laboratory of Child Development and Disorders, Children’s Hospital of Chongqing Medical University, Chongqing, China

**Keywords:** myelin oligodendrocyte glycoprotein, antibody, myelitis, magnetic resonance imaging, H-sign

## Abstract

Transverse myelitis is the second most common symptoms in myelin oligodendrocyte antibody-associated diseases (MOGAD), causing obvious clinical manifestation. T2-hyperintense lesions mainly restricted to the gray matter in the spinal cord on axial magnetic resonance imaging, produce the H-sign, which is thought to be the typical finding of MOGAD. Contrast enhancement can be observed in some cases of myelin oligodendrocyte antibody-associated transverse myelitis (MOG-TM). However, reports on the enhancement pattern associated with the H-sign are rarely seen. In this report, we describe a case of pediatric MOG-TM in which the H-sign was observed without enhancement, while the surrounding white matter exhibited enhancement. This pattern contradicts the previously observed gray matter involvement. Then we reviewed the literatures of myelin oligodendrocyte antibody-positive myelitis to focus on the neuroimaging features and discuss the implications of our finding.

## Introduction

Myelin oligodendrocyte antibody-associated disease (MOGAD) is a central nervous system (CNS) demyelinating disease that has been identified in recent years. Transverse myelitis (TM) is the second most common presentation, occurring in approximately 26% of MOGAD (1). TM is characterized clinically by an acute or subacute onset of motor, sensory, and autonomic symptoms and signs attributable to cord dysfunction (2). The severity of the attack varies, typically moderate to severe with expanded disability status scale (EDSS) scores >4 in about half of patients with up to one-third being non-ambulatory at the nadir of acute myelitis attack (3, 4), and many requiring bladder catheterization (5). Magnetic resonance imaging (MRI) is incorporated into the diagnostic criteria for MOGAD as the sole imaging modality capable of visualizing spinal cord lesions.

According to the latest diagnosis criteria of MOGAD (2023) (6), acute T2-hyperintense lesions restricted to the gray matter in the spinal cord on axial imaging, produce the H-sign (as seen in 30%–50% of patients), which is thought to be the typical finding of myelin oligodendrocyte antibody-associated transverse myelitis (MOG-TM) (6). This finding challenges the prevailing notion that demyelinating diseases primarily affect white matter in the central nervous system. Contrast enhancement is seen in approximately 50% of patients with MOG-TM, and cauda equina and pial enhancement have been reported (3, 7–9). Reports on the enhancement pattern associated with the H-sign are rarely seen.

MOG-TM can also be involved in children, who may be observed the H-sign on MRI. In this study, we presented one pediatric patient diagnosed with MOG-TM, observed a characteristic H-sign on axial MRI. Interestingly, only the white matter surrounding the H-sign exhibited enhancement.

## Case presentation

An 11-year-old boy exhibited progressive numbness and weakness in both lower extremities for 2 days after experiencing an unexplained fall 6 days prior to hospitalization (February 8, 2018). Within 2 days of admission, he experienced quadriplegia, in addition to sensory and motor deficits below the nipples bilaterally, as well as dysfunction of bowel and bladder control. Neurological and physical examinations demonstrated reduced muscle strength in the lower limbs, as well as the absence of knee, abdominal wall and cremasteric reflexes. His medical and family history were found to be unremarkable. Upon admission, the blood analysis showed an increase in white blood cell count (15.92 × 10^9^/L), an elevated percentage of neutrophils (93%), and an elevated level of C-reactive protein (11 mg/L). The following day, there was an increase in the total cellular count in the cerebrospinal fluid (CSF) (550 × 10^9^/L) and an increase in nucleated cells (420 × 10^6^/L). Serum and CSF samples were examined for demyelinating and autoimmune antibodies using a cell-based assay with full-length human antigenic substrates at the China branch of Euroimmun Medical Diagnostic Laboratory (EUROIMMUN AG, Lübeck, Germany). The results showed a positive titer of MOG-antibody at 1:100 in the serum and 1:1 in the CSF. However, other demyelinating antibodies such as aquaporin-4 (AQP-4) and autoimmune antibodies in both the blood and CSF tested negative.

**Figure 1:**
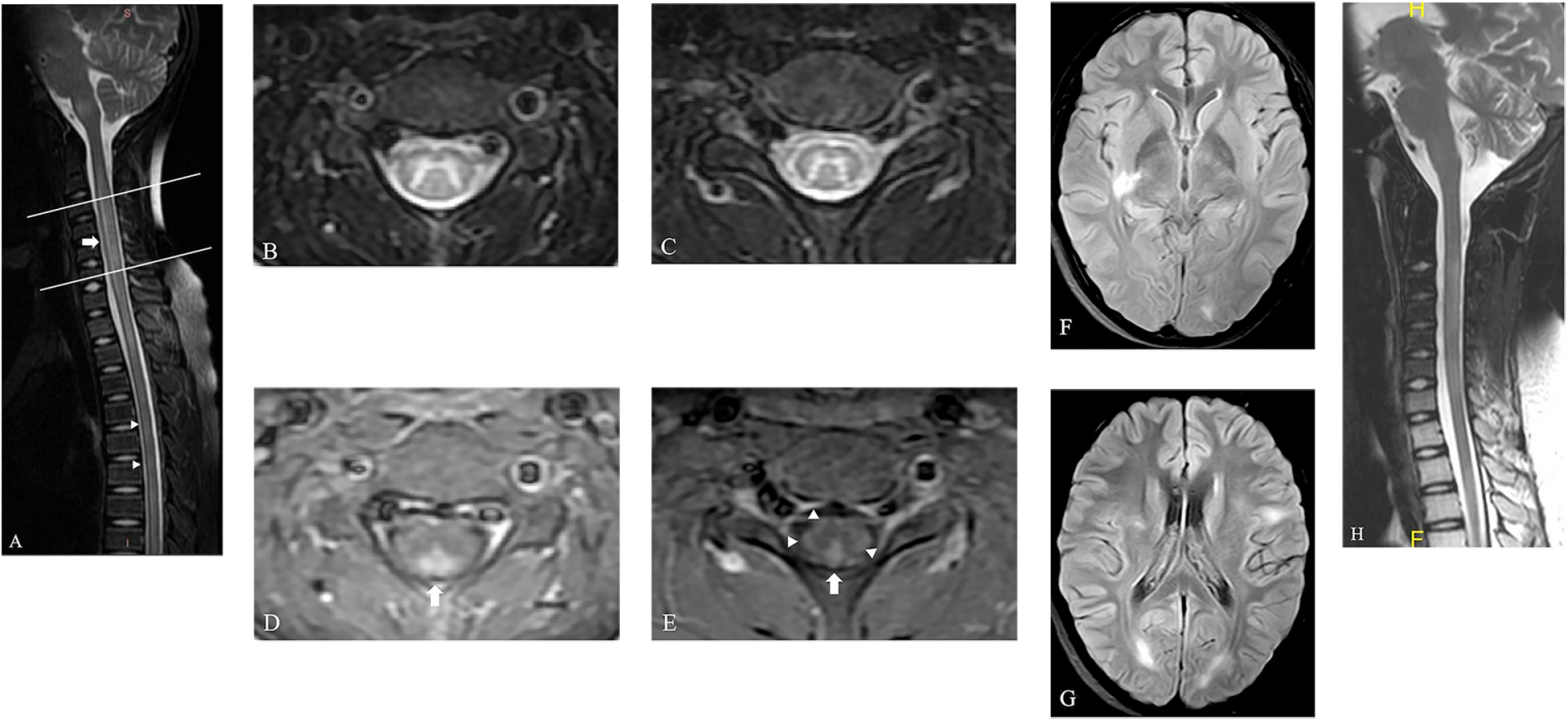
The spinal MRI of this patient. **(**A**)** The sagittal T2W image of spine shows LETM ranging from C2 to T1 segments (arrow) and SETM in thoracic spinal cord (arrow head). **(B,C)** Axial T2W images of spine show hyperintensity primarily located in the gray matter and displaying the characteristic “H-sign” in the level of C3 (the upper line in a) and C6 (the lower line in a). **(D,E)** This hyperintensity displays no enhancement, but the bilateral posterior funiculus was obviously enhanced on axial T1W images (arrow). The bilateral lateral funiculus and right anterior funiculus were also found moderate enhancement (arrow head). **(F,G)** Axial fluid-attenuated inversion recovery images of brain show multiple lesions located in the right basal ganglia and bilateral cerebral hemispheres, with no enhancement (not shown). **(H)** A follow-up MRI scan of spine shows obvious absorption of the previously abnormal signals in the spinal cord.

Six days after admission, a spinal MRI scan showed significant signal changes in the entire cervical and thoracic spinal cord segments on T1-weighted (T1W) and T2-weighted (T2W) sequences. These changes were consistent with a diagnosis of longitudinally extensive transverse myelitis (LETM) and short transverse myelitis (SETM) (Figure 1A), with the C2-T6 segment primarily affected in the gray matter and displaying the characteristic “H-sign” (Figures 1B,C). The white matter exhibited pronounced enhancement following the injection of contrast agent, particularly in the posterior funiculus, depicting primarily patchy enhancement. However, no enhancement was detected in the H-sign (Figures 1D,E). After performing a cranial MRI scan, multiple lesions were observed in the bilateral cerebral hemispheres, right basal ganglia, pons, and medulla, without any enhancement (Figures 1F,G).

Therefore, the diagnosis of MOGAD was confirmed, and first-line immunotherapy, including intravenous methylprednisolone and immunoglobulin, was commenced promptly. After 2 months of consistent treatment, there was a marked improvement in symptoms, with only residual symptoms remaining, such as mild bladder dysfunction and motor dysfunction in both lower limbs. The oral steroid dosage was taken for about 40 days and subsequently tapered gradually with outpatient monitoring. After 41 days since the initial scan, a follow-up MRI revealed obvious absorption of the previously abnormal signals in the spinal cord without any noticeable enhancement, consistent with the clinical presentation (Figure 1H). During the latest telephone follow-up on April 8, 2024, his family reported no recurrence of symptoms.

### Literature review on neuroimaging

We then performed a review of current literatures, using “myelin oligodendrocyte glycoprotein” or “MOG” as the search terms, to identify research studies performed on myelin oligodendrocyte antibody-positive myelitis focusing on the description of the “H-sign” and enhancement pattern. Studies lacking MRI original data were excluded. Data of 237 patients were collected from eight publications and Table 1 summarizes 135 cases of MOG-TM with the H-sign, accounting for 29.4%–83.3% of the incidence rate. The proportion of lesion enhancement also varied, ranging from 25.0% to 60.0%. The enhancement pattern were typically patchy and faint or nodular. Moreover, leptomeningeal or subpial enhancement and spinal nerve roots enhancement were not uncommon. In three pediatric cohorts (total *n* = 77), the H-sign were observed in 51 out of 77 cases (66.2%). 50.0% (26/52) lesion enhancement were found in children with MOG-antibody-positive myelitis, which is the most common. This is then followed by the enhancement of leptomeninges and nerve roots, making up 38.5% (20/52) and 30.8% (16/52) respectively. Unfortunately, there was insufficient information available regarding the enhancement of lesions displaying the H-sign.

**Table 1:** A review of patients of MOGAD with H sign published in the last 10 years.

No. /Total no. (%)	No. 1 mixed cohort	No. 2 mixed cohort	No. 3 mixed cohort	No. 4 pediatric cohort	No. 5 mixed cohort	No. 6 mixed corhort	No. 7 pediatric corhort	No. 8 pediatric corhort
Research	Dubey et al. (3)	Zhang Bao et al. (10)	Fadda et al. (9)	Tantsis et al. (2)	Kitley et al. (11)	Netravathi et al. (12)	El Naggar et al. (13)	Ren et al. (14)
Demographics (*n*=)	54	38	40	10	9	19	33	34
Typical age at first myelitis (year)	3–73 (median: 25)	7–57 (mean: 28.13 ± 12.74)	3.80–11.48 (median: 6.71)	6.0–14.8 (median:11.0)	mean: 32.29	1–66 (median:21)^d^	5–10 (median: 7)	0.5–13.1 (7)
Sex (F:M)	24:30	62:68	23:17	6:4	4:5	13:6	14:19	16:18
Follow-up time	2–120 months (median: 24)	NA	4.14–8.30 years (median: 5.96)	NA	22–38.5 months (median: 18)	median: 1 year	NA	8–109 months (median: 28)
Clinical features								
Antecedent	31 (57%)	23/55^a^ (41.8%)	NA	NA	NA	40/93 (43.0%)	NA	3/35^a^ (8.6%)
Infection/immunization	33 (61%)	3/55^a^ (5.5%)	NA	NA	NA	0	NA	1/35^a^ (2.7%)
Disease course (monophasic or relapsing)	Relapsing (32/54, 59.3%）	Relapsing (75/130, 57.7%）	NA	NA	Monophasic	Relapsing (3/19, 15.8%)	NA	Relapsing (6/34, 17.7%）
Myelitis symptoms								
Weakness	45/54 (83.3)	29/38 (76.3)	NA	10/10 (100.0)	NA	NA	NA	32/35 (91.4)
Sensory disturbances	48/54 (88.9)	33/38 (86.8)	NA	8/10 (80.0)	NA	NA	NA	16/35 (45.7)
Bowel/bladder dysfunction	45/54 (83.3)	26/38 (68.4)	NA	9/10 (90.0)	33%	NA	NA	22/35 (62.9)
Erectile dysfunction in men	13/24 (54.2)	NA	NA	NA	NA	NA	NA	NA
EDSS ≥ 7 at myelitis nadir	18/54 (33.3)	NA	NA	NA	Median 6.0 (range: 4–8.5)	NA	NA	16/35 (45.7)
Recovery from myelitis attacks	Median mRS was 1 (range: 0–4)	Median EDSS was 1 (range, 0–7.5)	Median EDSS was 1 (range, 0–1.5) in 22 patients	NA	The median EDSS was 0 (range, 0–2.5)	NA	NA	Median EDSS was 0 (range, 0–2)
Need for gait aid long term	3/54 (5.6）	NA	0/22^b^	NA	0	NA	NA	0
Extra spine manifestations								
Optic neuritis	NA	11/27 (40.7)	2/40 (5.0)	NA	4/9 (44.4)	NA	7/33 (21.2)	NA
Encephalopathy	✓		17/40 (42.5)	NA	2/9 (22.2)	NA	14/33 (42.4)	15/35 (42.9)
Non-specific symptoms	NA	NA	6/40 (15.0)	NA	NA	NA	NA	NA
CSF findings acutely								
Oligoclonal bands	1/38 (2.6)	NA	NA	0/8	4/9 (44.4)	3 (15.8%)	4/32 (12.5)	11/35 (31.4)
White cell count >5/µl	30/42 (71.4)	NA	NA	NA	>10/ul: 5（55.6%)	8 (42.1%)	NA	22/35 (62.9)
White cell count >50/µl	22/42 (52.4)	NA	NA	NA	NA		NA	NA
Spinal MRI findings at onset								
Negative	3	NA	1	NA	0	NA	0	NA
Positive (T2 hyper intensity)	51	NA	39	NA	9	41	33/33	NA
Axial H sign	15/51 (29.4)	26/52 (50.0)	22/35 (62.9)	5/10 (50.0)	5/6 (83.3)	16^c^	24/33 (72.7)	22/35 (62.9)
Enhancement pattern	14/54 (25.9%) lesion enhancement, patchy and faint	NA	Nodular 8/32 (25.0%), leptomeningeal enhancement 22/32 (68.8%), spinal roots 11/32 (34.4%)	Lesion enhancement 2/8 (25.0%), ventral nerve root enhancement 2/8 (25.0%), dorsal nerve root enhancement 2/8 (25.0%)	2/6 (33.3%) lesion enhancement, details NA	Lesion enhancement (*n* = 19) and subpial enhancement (*n* = 5, 12.2%)	Lesion enhancement 18/30 (60.0%), leptomeningeal enhancement 16/30 (53.3%), Nerve root enhancement 4/30 (13.3%)	Lesion enhancement 6/14 patients (42.9%), typically patchy and faint. leptomeningeal enhancement 4/14 (28.6%), spinal roots enhancement 8/14 (57.1%)

^a^ Total numbers of myelitis attacks.

^b^ 22 cases had EDSS scores.

^c^ Total of lesions was not available.

^d^ Total numbers of MOGAD, not myelitis.

## Discussion

Studies from multiple countries support MOGAD as a global disease affecting people of all ages. The incidence of MOGAD is 1.6–3.4 per million people per year, and the prevalence is estimated at 20 per million (95% CI 11–34) (15, 16). Myelin oligodendrocyte glycoprotein (MOG) is expressed on the surface of the myelin sheath, mainly in CNS neurons, and accounts for about 0.5% of myelin components (17, 18). Thus, MOGAD is generally considered to have a favorable prognosis compared to other CNS demyelinating diseases such as AQP-4 positive neuromyelitis optica spectrum disorder (NMOSD) and multiple sclerosis (MS). Nevertheless, MOGAD have garnered significant interest among neurologists due to its tendency to relapse.

TM, which is one of the main symptoms of MOGAD, has an incidence of roughly 20%–30%, a male-to-female ratio of roughly 1:1, and a peak age of onset at 20–40 years old (10). Younger children usually present as a component of acute disseminated encephalomyelitis (ADEM), while older children may be concurrent with optic neuritis (12). Most patients experience good to excellent motor recovery (3, 7, 19), but permanent bladder, bowel or sexual dysfunction can occur (3, 7, 20). Early detection of lesions, especially the typical findings of MOG-TM on MRI, facilitates early immune intervention. According to the latest criteria proposed by the International MOGAD Panel, the presence of H-sign on MRI is considered a supportive feature for the diagnosis of MOGAD in cases of myelitis (6).

We report a pediatric patient diagnosed with MOGAD who had the characteristic H-sign on spinal MRI. It is widely accepted that the enhancement of lesions in MOGAD on MRI is thought to be due to the disruption of the blood-brain barrier (BBB) resulting from inflammation. Circulating cytokines may increase the BBB's permeability and promote inflammatory infiltration into the CNS (21, 22). In this case, the T2 hyperintensity was identified in the gray matter (referred to as the H-sign) without any enhancement. In contrast, the surrounding white matter displayed significant enhancement. This suggests that the BBB in the white matter may have been compromised due to inflammation, despite the absence of hyperintensity on T2W images. Therefore, the pathological alterations may not be restricted solely to the gray matter exhibiting T2 hyperintensity, but also involve the white matter with potentially more severe effects. Our results did not align with previous studies suggesting that the presence of the H-sign on MRI indicated exclusive or primary gray matter involvement in MOG-TM (2–4, 23).

Due to the important role of the H-sign in diagnosis, a review of literatures of MOG-TM cases with the H-sign from the last decade was undertaken to gain insight into its etiology. A total of 135 patients of MOG-TM displaying the H-sign were collected from eight studies, in which only three were pediatric cohort, the other five were mixed cohort. The most common clinical manifestations reported in these eight studies were weakness, sensory disturbances and bowel/bladder dysfunction. Although some patients with MOG-TM experienced severe symptoms (EDSS ≥ 7) at onset, the majority of patients had complete resolution of symptoms during follow-up, with residual sphincter dysfunction in some cases. These results are generally in accordance with mild myelin damage observed in the pathology of MOGAD (24). Simultaneously, we further summarize the enhancement characteristics of these cases. The H-sign was reported ranging from 29.4% to 83.3% and was also common in pediatrics accounting for approximately 66.2%. In terms of enhancement, there were lesions with enhancement (typically patchy and faint or nodular), leptomeningeal or subpial enhancement and spinal nerve root enhancement. Approximately half of the pediatric patients displayed focal enhancement of the lesion, however, the specific details of the enhancement in relation to the H-sign were not clarified.

The findings from prior studies have confirmed that a large proportion of individuals diagnosed with MOG-TM demonstrate a positive outcome, regardless of the severity of symptoms present at the disease onset (25). This may be attributed to the lesser impact of the disease on gray matter, which is a crucial organ housing neurons, and is not the primary site of damage in demyelinating diseases. Therefore, in conjunction with our case, we propose that although the gray matter exhibits hyperintensity on T2W images (appearing as the H-sign) and seems to be the most severely affected area, the lesions may primarily consist of edema and the BBB remains intact. Once the inflammation resolves, the edema would decrease soon and the clinical manifestations would vanish. However, the white matter, which did not display obvious hyperintensity adjacent to the gray matter on T2W images, showed enhancement, indicating a disruption of the BBB. Nevertheless, the demyelination process of the white matter appeared to be more amenable to repair than the gray matter damage. The difference of enhancement pattern between the gray and white matter might be a possible reason to explain the positive outcome of MOG-TM.

As previously stated, most of MOG-TM lesions tend to resolve effectively, leading to a limited number of spinal cord biopsies available to substantiate our findings. According to a recent animal study on MOG-IgG experimental autoimmune encephalomyelitis (EAE), inflammation was found to initiate primarily in the white matter region during both acute and chronic stages of the disease (26). The infiltration and activation of inflammatory cells such as macrophages and T-cells mainly existed in the white matter with a small amount in the gray matter. The rate of spinal demyelinating lesions in these model mice was relatively high, particularly in the posterior funiculus, where it exceeded 60% at the acute stage (26). Thus, we speculate that the possible cause of the enhancement of the posterior funiculus, located opposite to the H-sign area in our case, may be attributed to the accumulation of MOG antibodies or a significant presence of inflammatory cells in the white matter. In the acute stage of MOGAD, there should be inflammatory edema present in both white and gray matter, leading to increased T2W signals in both areas. This is consistent with MOG-TM lacking the characteristic H-sign. However, the high levels of MOG antigen in the white matter region (27), may lead to a concentration of MOG antibody or inflammatory cells, potentially reducing the T2W signal in the white matter and eventually causing the formation of the H-sign. The elevated cellularity or high concentration deposition of protein may lead to a decrease in the T2W signal within the lesion area, as demonstrated in various other medical conditions (28–30). Additional animal experiments and clinical studies are necessary to validate the relationship between the H sign and the levels of MOG antibodies in MOGAD. Nevertheless, our findings indicate that it is imperative to not only prioritize gray matter lesions in MOG-TM due to the presence of the H sign, but also to consider the possibility of more severe lesions in the white matter.

There are several limitations to our study. First, our sample size utilized was limited, and studies with larger sample is needed to clarify the enhancement pattern of the H-sign. Second, pathological biopsy evidence was lacking and only animal experiments were conducted to support the findings. Third, prospective experiments are required to investigate the correlation between the H-sign and serum antibody titers.

This study presents a pediatric case of MOG-TM, who exhibited a distinct H-sign and an unusual enhancement pattern. It is important to highlight that the H-sign, indicative of gray matter involvement, may not represent the critical anatomical region associated with MOG-TM. Furthermore, we recommend the use of enhanced MRI for patients suspected of having MOG-TM.

## References

[1] Longbrake E. Myelin oligodendrocyte glycoprotein-associated disorders. Contin Lifelong Learn Neurol. (2022) 28:1171–93. 10.1212/CON.0000000000001127 PMC9523511 35938661

[2] Tantsis EM Prelog K Alper G Benson L Gorman M Lim M Magnetic resonance imaging in enterovirus-71, myelin oligodendrocyte glycoprotein antibody, aquaporin-4 antibody, and multiple sclerosis-associated myelitis in children. Dev Med Child Neurol. (2019) 61(9):1108–16. 10.1111/dmcn.14114 30537075

[3] Dubey D Pittock SJ Krecke KN Morris PP Sechi E Zalewski NL Clinical, radiologic, and prognostic features of myelitis associated with myelin oligodendrocyte glycoprotein autoantibody. JAMA Neurol. (2019) 76(3):301–9. 10.1001/jamaneurol.2018.4053 30575890 PMC6440233

[4] Mariano R Messina S Roca-Fernandez A Leite MI Kong Y Palace JA. Quantitative spinal cord MRI in MOG-antibody disease, neuromyelitis optica and multiple sclerosis. Brain. (2021) 144(1):198–212. 10.1093/brain/awaa347 33206944

[5] Sechi E Cacciaguerra L Chen JJ Mariotto S Fadda G Dinoto A Myelin oligodendrocyte glycoprotein antibody-associated disease (MOGAD): a review of clinical and MRI features, diagnosis, and management. Front Neurol. (2022) 13(885218). 10.3389/fneur.2022.885218 PMC9247462 35785363

[6] Banwell B Bennett JL Marignier R Kim HJ Brilot F Flanagan EP Diagnosis of myelin oligodendrocyte glycoprotein antibody-associated disease: international MOGAD panel proposed criteria. Lancet Neurol. (2023) 22(3):268–82. 10.1016/S1474-4422(22)00431-8 36706773

[7] Mariano R Messina S Kumar K Kuker W Leite MI Palace J. Comparison of clinical outcomes of transverse myelitis among adults with myelin oligodendrocyte glycoprotein antibody vs aquaporin-4 antibody disease. JAMA Netw Open. (2019) 2(10):e1912732. 10.1001/jamaneurol.2018.4053 31596489 PMC6802235

[8] Rinaldi S Davies A Fehmi J Beadnall HN Wang J Hardy TA Overlapping central and peripheral nervous system syndromes in MOG antibody-associated disorders. Neurol Neuroimmunol Neuroinflamm. (2020) 8(1):e924. 10.1212/NXI.0000000000000924 33272955 PMC7803332

[9] Fadda G Alves CA O’Mahony J Castro DA Yeh EA Marrie RA Comparison of spinal cord magnetic resonance imaging features among children with acquired demyelinating syndromes. JAMA Netw Open. (2021) 4(10):e2128871. 10.1001/jamanetworkopen.2021.28871 34643718 PMC8515204

[10] ZhangBao J Huang W Zhou L Wang L Chang X Lu C Myelitis in inflammatory disorders associated with myelin oligodendrocyte glycoprotein antibody and aquaporin-4 antibody: a comparative study in Chinese Han patients. Eur J Neurol (2021) 28(4):1308–15. 10.1111/ene.14654 33220172

[11] Kitley J Waters P Woodhall M Leite MI Murchison A George J Neuromyelitis optica spectrum disorders with aquaporin-4 and myelin-oligodendrocyte glycoprotein antibodies: a comparative study. JAMA Neurol. (2014) 71(3):276–83. 10.1001/jamaneurol.2013.5857 24425068

[12] Netravathi M Holla VV Nalini A Yadav R Vengalil S Oommen AT Myelin oligodendrocyte glycoprotein-antibody-associated disorder: a new inflammatory CNS demyelinating disorder. J Neurol. (2021) 268(4):1419–33. 10.1007/s00415-020-10300-z 33188477

[13] Naggar IE Cleaveland R Wendel EM Bertolini A Schanda K Karenfort M MR imaging in children with transverse myelitis and acquired demyelinating syndromes. Mult Scler Relat Disord. (2022) 67(104068). 10.1016/j.msard.2022.104068 35933757

[14] Ren C Zhang W Zhou A Zhou J Cheng H Tang X Clinical and radiologic features among children with myelin oligodendrocyte glycoprotein antibody-associated myelitis. Pediatr Neurol. (2023) 143:96–9. 10.1016/j.pediatrneurol.2023.02.019 37060644

[15] de Mol CL Wong Y van Pelt ED Wokke B Siepman T Neuteboom RF The clinical spectrum and incidence of anti-MOG-associated acquired demyelinating syndromes in children and adults. Mult Scler. (2020) 26(7):806–14. 10.1177/1352458519845112 31094288 PMC7294530

[16] O’Connell K Hamilton-Shield A Woodhall M Messina S Mariano R Waters P Prevalence and incidence of neuromyelitis optica spectrum disorder, aquaporin-4 antibody-positive NMOSD and MOG antibody-positive disease in Oxfordshire, UK. J Neurol Neurosurg Psychiatry. (2020) 91(10):1126–8. 10.1136/jnnp-2020-323158 32576617

[17] Reindl M Waters P. Myelin oligodendrocyte glycoprotein antibodies in neurological disease. Nat Rev Neurol. (2019) 15(2):89–102. 10.1038/s41582-018-0112-x 30559466

[18] Pham-Dinh D Mattei MG Nussbaum JL Roussel G Pontarotti P Roeckel N Myelin/oligodendrocyte glycoprotein is a member of a subset of the immunoglobulin superfamily encoded within the major histocompatibility complex. Proc Natl Acad Sci U S A. (1993) 90(17):7990–4. 10.1073/pnas.90.17.7990 8367453 PMC47273

[19] Ciron J Cobo-Calvo A Audoin B Bourre B Brassat D Cohen M Frequency and characteristics of short versus longitudinally extensive myelitis in adults with MOG antibodies: a retrospective multicentric study. Mult Scler. (2020) 26(8):936–44. 10.1177/1352458519849511 31148523

[20] Jurynczyk M Messina S Woodhall MR Raza N Everett R Roca-Fernandez A Clinical presentation and prognosis in MOG-antibody disease: a UK study. Brain. (2017) 140(12):3128–38. 10.1093/brain/awx276 29136091

[21] Corbali O Chitnis T. Pathophysiology of myelin oligodendrocyte glycoprotein antibody disease. Front Neurol. (2023) 14(1137998). 10.3389/fneur.2023.1137998 36925938 PMC10011114

[22] McCusker RH Kelley KW. Immune-neural connections: how the immune system’s response to infectious agents influences behavior. J Exp Biol. (2013) 216(Pt 1):84–98. 10.1242/jeb.073411 23225871 PMC3515033

[23] Mishal B Divate P. ‘H’ sign in a case of MOG myelitis. Neurol India. (2022) 70(4):1682–3. 10.4103/0028-3886.355180 36076688

[24] Takai Y Misu T Fujihara K Aoki M. Pathology of myelin oligodendrocyte glycoprotein antibody-associated disease: a comparison with multiple sclerosis and aquaporin 4 antibody-positive neuromyelitis optica spectrum disorders. Front Neurol. (2023) 14(1209749). 10.3389/fneur.2023.1209749 37545724 PMC10400774

[25] Jarius S Ruprecht K Kleiter I Borisow N Asgari N Pitarokoili K MOG-IgG in NMO and related disorders: a multicenter study of 50 patients. Part 2: epidemiology, clinical presentation, radiological and laboratory features, treatment responses, and long-term outcome. J Neuroinflammation. (2016) 13(1):280. 10.1186/s12974-016-0718-0 27793206 PMC5086042

[26] Remlinger J Bagnoud M Meli I Massy M Linington C Chan A Modelling MOG antibody-associated disorder and neuromyelitis optica spectrum disorder in animal models: spinal cord manifestations. Mult Scler Relat Disord. (2023) 78(104892). 10.1016/j.msard.2023.104892 37499337 PMC11792092

[27] Mills JD Kavanagh T Kim WS Chen BJ Kawahara Y Halliday GM Unique transcriptome patterns of the white and grey matter corroborate structural and functional heterogeneity in the human frontal lobe. PLoS One. (2013) 8(10):e78480. 10.1371/journal.pone.0078480 24194939 PMC3808538

[28] Fujita A Sakai O Chapman MN Sugimoto H. IgG4-related disease of the head and neck: CT and MR imaging manifestations. Radiographics. (2012) 32(7):1945–58. 10.1148/rg.327125032 23150850

[29] Baur A Stäbler A Lamerz R Bartl R Reiser M. Light chain deposition disease in multiple myeloma: MR imaging features correlated with histopathological findings. Skeletal Radiol. (1998) 27(3):173–6. 10.1007/s002560050360 9554011

[30] Wang Q Takashima S Fukuda H Takayama F Kobayashi S Sone S. Detection of medullary thyroid carcinoma and regional lymph node metastases by magnetic resonance imaging. Arch Otolaryngol Head Neck Surg. (1999) 125(8):842–8. 10.1001/archotol.125.8.842 10448729

